# Immediate aortic dissection after transcatheter aortic valve replacement: A case report and review of the literature

**DOI:** 10.1002/ccr3.4412

**Published:** 2021-07-06

**Authors:** Pongprueth Rujirachun, Apichaya Junyavoraluk, Decho Jakrapanichakul, Nattawut Wongpraparut, Narathip Chunhamaneewat, Adisak Maneesai, Pranya Sakiyalak

**Affiliations:** ^1^ Department of Microbiology Faculty of Medicine Siriraj Hospital Mahidol University Bangkok Thailand; ^2^ Department of Surgery Faculty of Medicine Khon Kaen University Khon Kaen Thailand; ^3^ Division of Cardiology Department of Medicine Faculty of Medicine Siriraj Hospital Mahidol University Bangkok Thailand; ^4^ Division of Cardiovascular Thoracic Surgery Department of Surgery Faculty of Medicine Siriraj Hospital Mahidol University Bangkok Thailand

**Keywords:** acute complication, aortic dissection, thoracic endovascular aortic repair (TEVAR), transcatheter aortic valve replacement (TAVR), transesophageal echocardiogram (TEE)

## Abstract

‐Aortic dissection of descending aorta was detected by intraoperative TEE in a case of 67‐year‐old man with symptomatic severe aortic stenosis after TAVR.

‐Transesophageal echocardiogram after TAVR procedure is helpful to detect this rare complication.

## Graphical abstract

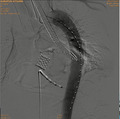

## 1 INTRODUCTION

We report a severe aortic stenosis patient treated by TAVR. After the replacement valve was deployed, we found abnormal leakage with flap at the descending aorta by transesophageal echocardiogram. Under intraoperative close monitoring, the dissection was found to be expanding so we decided to perform thoracic endovascular aortic repair.

Transcatheter aortic valve replacement (TAVR) can cause potential complications, including vascular injury, paravalvular leakage, valve migration, conduction disturbances, embolic stroke, coronary obstruction, and aortic dissection. Iatrogenic aortic dissection is a rare but highly serious complication.^1^ However, it can be detected and treated early. Due to the risk of aortic rupture, acute ascending aortic dissection is usually treated by emergency surgical repair, while descending aortic dissection is likely to be treated conservatively, except for complicated cases with impending rupture, uncontrolled pain, malperfusion, and uncontrolled blood pressure, which must be treated by endovascular therapy.^2^

## 2 CASE PRESENTATION

A 67‐year‐old man diagnosed as symptomatic severe aortic stenosis (aortic valve area [AVA] of 0.67 cm^2^ and mean pressure gradient [mPG] of 27 mm Hg) was referred to our hospital for consideration of TAVR. He had a medical history of type 2 diabetes mellitus, end‐stage renal disease, for which he had been on hemodialysis for 13 years, triple‐vessel disease postcoronary artery bypass grafting for 7 years, and was categorized as New York Heart Association functional class III. The Society of Thoracic Surgeons' risk of mortality and Logistic EuroSCORE II for surgical aortic valve replacement scores were 28.32% and 22.65%, respectively.

Coronary angiography showed triple‐vessel disease, while bypass graft angiography revealed 80% stenosis at the distal one‐third of the Y‐graft or saphenous vein graft to the obtuse marginal and diagonal branch graft (SVG‐OM/DG).

After discussing the case in our heart team, the patient was determined to be a high‐risk patient for surgery, and consequently, the consensus was reached to perform TAVR promptly with percutaneous coronary intervention (PCI), together with detailed preoperative evaluation using multimodality imaging tools.

Transesophageal echocardiography (TEE) was performed and revealed heavy calcification and fusion of the noncoronary cusp and left coronary cusp of the aortic valve (compatible with bicuspid aortic valve) with a left ventricle ejection fraction (LVEF) of 37%. The aortic mPG was 29.89 mm Hg, the peak flow velocity through the aortic valve was 3.81 m/s, the stroke volume was 54.12 mL at the left ventricular outflow tract, and the aortic valve area (AVA) was calculated to be 0.81 mm^2^, compatible with low‐flow, low‐gradient, severe aortic stenosis (AS). It should be noted that dobutamine stress echocardiography was avoided because the patient had ischemic cardiomyopathy so severe aortic stenosis was diagnosed based on the severe aortic calcification.

**FIGURE 1:**
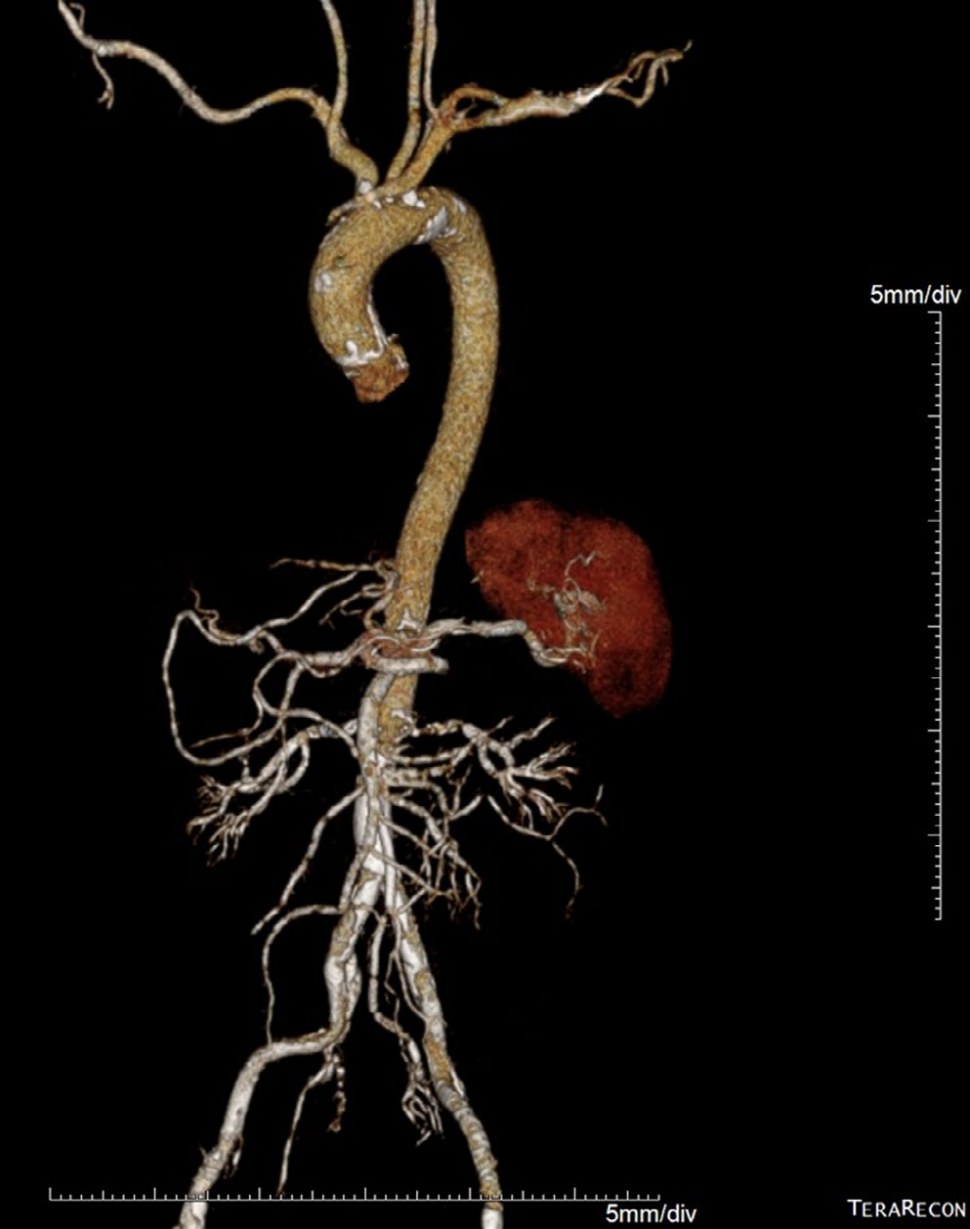
Three‐dimensional CTA showing scattered plaques with calcification of the ascending aorta without aortic dissection and stenosis before TAVR

Computed tomography angiography (CTA) revealed an aortic valve annulus area of 3.80 cm^2^ (diameter 22 mm) together with a trileaflet aortic valve with heavy calcification with a score of 2000 Agatston units (AU) and a mitral valve with mild calcification. The left main and right coronary heights were 13.8 and 13.6 mm, respectively. Scattered calcified plaques were found on the ascending aorta (Figure 1). The right and left common femoral artery diameters were 7.2 and 6.8 mm, respectively, and there was an arteriovenous (AV) fistula at the left common femoral artery with dense calcification at the level of the femoral head. The abdominal aorta was moderately calcified, with no dissection and no stenosis along the entire aorta. The right femoral approach was considered more suitable for TAVR with a 23 mm Edwards SAPIEN 3 transcatheter heart valve (Edwards Lifesciences Corporation).

**FIGURE 2:**
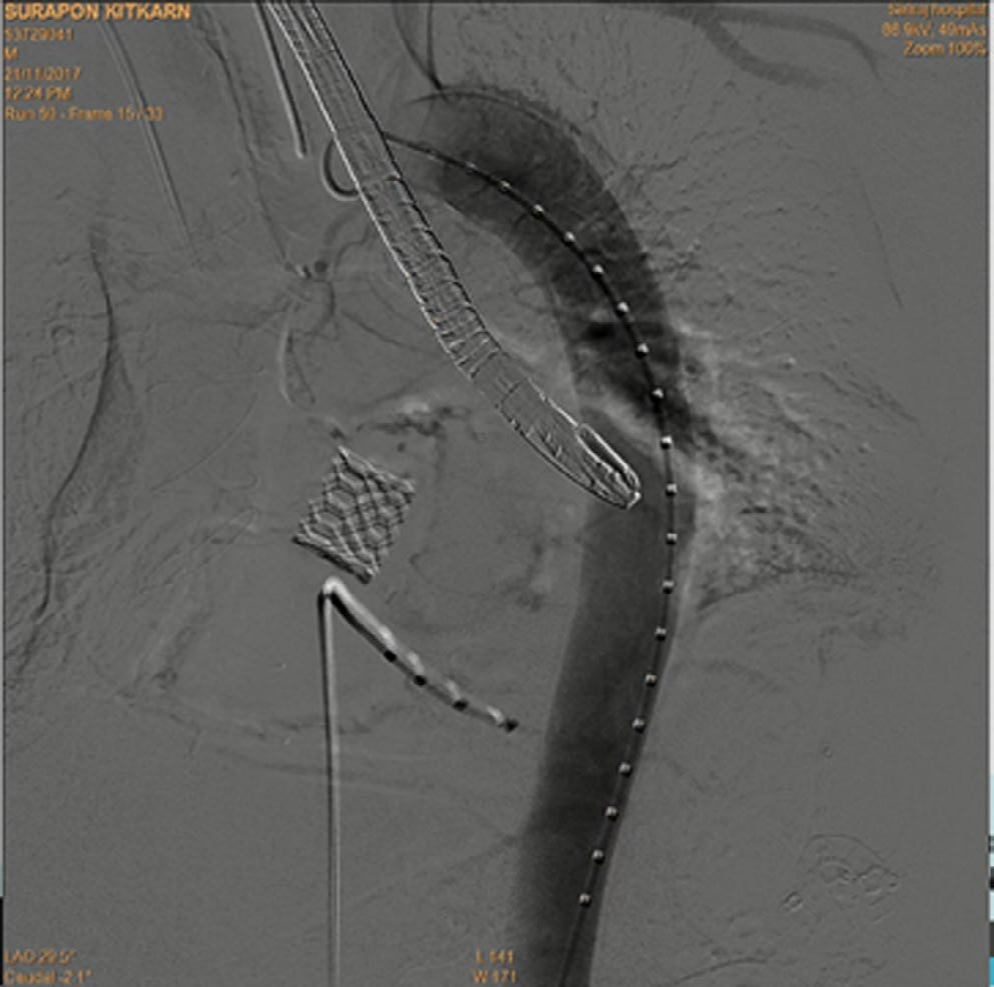
Angiogram after TAVR showing no evidence of dissection or rupture at the thoracic aorta. The previously implanted aortic valve can be well seen

**FIGURE 3:**
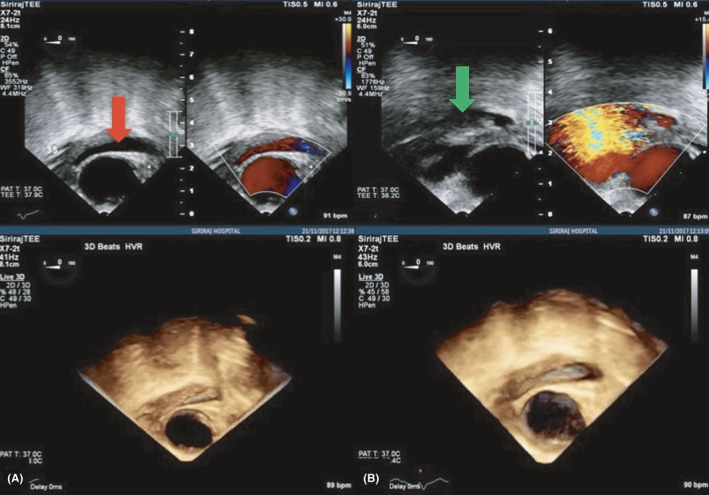
Intraoperative transesophageal echocardiogram after TAVR showing the further extent of the echo‐lucent area in the descending aorta by serial TEE from A (Red arrow) to B (Green arrow)

**FIGURE 4:**
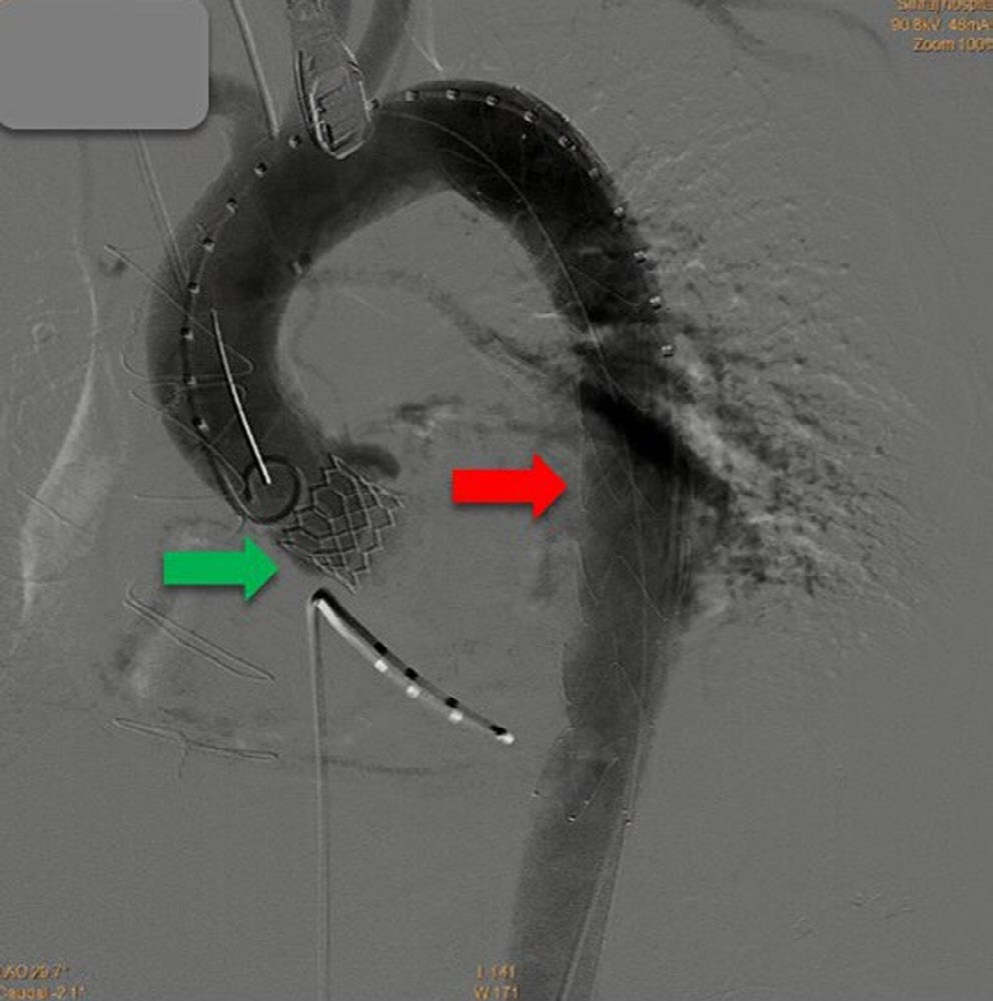
Final angiogram after stent graft deployment at the descending aorta (Red arrow). The previously implanted aortic valve can be well seen (Green arrow)

Transcatheter aortic valve replacement was performed under general anesthesia. The left femoral artery and vein were punctured and cannulated with a 6 Fr vascular sheath for pigtail insertion. The pacemaker was inserted via the left femoral vein. Percutaneous coronary intervention (PCI) was done with an Onyx stent (5 × 15 mm) in SVG‐OM/DG under fluoroscopic guidance. A guidewire was inserted from the right femoral artery and passed through the entire aorta and through the aortic valve, and then, balloon aortic valvuloplasty (diameter 20 mm) was done with the 23 mm Edwards SAPIEN 3 transcatheter heart valve under fluoroscopic guidance (Figure 2). After implantation of the valve, routine surveillance TEE showed a hypoechoic lesion around the descending aorta, for which acute aortic dissection of the mid descending aorta was suspected with early aortic expansion, because there was a colored flow in the space corresponding with the aortic pulsatile flow. This was surrounded with hypoechoic content and was progressively enlarged. The maximal thickness was 0.7 cm at a 35 cm depth of the TEE probe within 10 minutes of insertion (Figure 3), so we immediately did thoracic endovascular aortic repair (TEVAR) with Zenith Alpha^™^ Thoracic Endovascular Graft Proximal components (diameter 30 mm, length 155 mm). Then, the patient was evaluated by a second final angiogram (Figure 4), which showed a patent coronary artery, intact ascending aorta, descending aorta, abdominal aorta, and both common iliac arteries. Consequently, the wound was closed and the patient was transferred to the critical care unit (CCU) in a stable condition.

Seven days after the operation, the patient's clinical symptoms were improved. Computed tomography angiography (CTA) for post‐TEVAR evaluation was planned 7 days later, but the patient was lost to follow‐up and subsequently died a few weeks after discharge at his home from an unknown cause of death. However, the early death, in this case, most likely is procedure‐related than anything else.

## 3 DISCUSSION

Among the major vascular complications, aortic dissection (AD) is extremely rare. Most lesions occur on the ascending part, while descending aortic dissection‐related TAVR is very uncommon. There are many proposed mechanisms for TAVR related to ascending aortic dissection, such as catheter manipulation, valve repositioning or retraction, and inadequate balloon predication.^3^ Unlike descending aortic dissection, the main mechanism for ascending aortic dissection is caused by catheter or wire trauma and vascular sheath insertion. In order to prevent this complication, preoperative, intraoperative, and postoperative prevention steps should be considered. In the preoperative step, we need to predict the vascular complication not only at the access site but also along the entire aorta by using any imaging modality. Intraoperatively, the vascular system can easily be injured by the catheter. During catheter insertion and retraction, the operator should keep the position of the sheath constant and place the introduced guidewire in the proper position under fluoroscopy guidance. In the postoperative step, we suggest doing TEE for detecting any issues or where there is any doubt for major vascular complication.

There is no guideline for the iatrogenic treatment of descending aortic dissection. In the case of a limited and uncomplicated dissection, conservative treatment could be applied. However, in complicated cases, like an impending rupture, uncontrolled pain, malperfusion, early aortic expansion, or uncontrolled blood pressure, TEVAR should be considered to reduce the mortality, morbidity, and paraplegia risk compared with a conventional open repair.^4^ In the present case, there was an early aortic expansion of the descending aortic dissection, which prompted our decision to perform TEVAR instead of conservative treatment.

We summarize the case reports in the literature of post‐TAVR aortic dissection from 2010 up until now inTable 1.^2^, ^4^, ^5^, ^6^, ^7^, ^8^, ^9^, ^10^, ^11^, ^12^, ^13^, ^14^ Most of the patients were women, and their ages were older than in our case. There are many arterial access techniques that can be used in TAVR, such as transfemoral, transapical, and transaortic access. From the table below, it can be seen that all the reported cases involved transfemoral or transaortic access. In transfemoral access, the guidewire passes through the femoral artery, abdominal aorta, descending aorta, ascending aorta, and aortic valve consecutively.^15^

**TABLE 1:** Reported cases of severe AS status post‐TAVR with aortic dissection

Author	Sex/Age	Comorbidity	Detection time of AD	Site of AD	Detection of AD	Treatment for AD	Result	Route access of TAVR
This case 2018	M/67	DM, ESRD	Immediate after implant	Ds	TEE	TEVAR	Died a few week	Femoral
Losmanova et al 2018^12^	F/81	NA	3 y	As	Autopsy	Conservative	Died	NR
Kilic et al 2017^14^	F/87	Previous CBG, heart failure, peripheral vascular disease	Immediate after implant	Ds	TEE	TEVAR	Recovery	Femoral
Kratimenos et al 2016^2^	F/81	COPD, renal dysfunction, angiodysplasia	12 d	Ds	CT	TEVAR	Recovery	Femoral
Nagasawa et al 2016^4^	F/86	Heart failure	During the procedure	Ds	TEE	Conservative	Recovery	Femoral
Yashima et al 2015^13^	F/88	NA	3 d	As	CT	Conservative	Recovery	Femoral
Van Mieghem et al 2013^5^	F/86	Coronary artery disease, posttotal knee prosthesis	1‐2 h	As	Angiogram	TEVAR	Recovery	Femoral
Loeser et al 2013^9^	F/89	NA	2‐5 h	As	Autopsy	NA	Cardiogenic shock and died	Femoral
Bibombe et al 2013^10^	M/83	Previous CBG, HT, DLP	During the procedure	As and Ds	TEE, CTA, angiogram	Open surgery	Recovery	Femoral
Al‐Attar et al 2013^11^	F/84	HT, thrombophlebitis	8 mo 2 wk	As	CT	Open surgery	Arrest and died	Femoral
D'Onofrio et al 2012^7^	F/79	RA, pulmonary edema, cerebral hemorrhage	Immediate after implant	As	TEE	Open surgery	Died 32 d later due to septic shock	Aortic
Ong et al 2011^6^	M/90	HT, CA prostate, CKD, gastric and duodenal ulcer	Immediate after implant	As	TEE	Conservative	Recovery	Femoral
Gerber et al 2010^8^	F/83	DM, LE	22 d	As	Autopsy	NA	Cardiac arrest and died	Femoral

Abbreviations: AD, Aortic dissection; As, Ascending; CA, cancer; CBG, Coronary bypass graft; CKD, Chronic kidney disease; COPD, Chronic obstructive pulmonary disease; DLP, Dyslipidemia; DM, Diabetes mellitus; Ds, Descending; ESRD, End‐stage renal disease; F, Female; HT, hypertension; LE, Lupus erythematosus; M, Male; NA, Not available; RA, Rheumatoid arthritis; TEVAR, Thoracic endovascular aortic repair.

The present case report describes the case of a male patient faced with unexpected acute descending aortic dissection after TAVR. Fortunately, our postoperative TAVR practice recommends doing TEE or aortogram in every case who is in doubt for aortic complication so such patients can be detected early, even if they show no signs or symptoms, so that they can be treated before transfer to the recovery room. Due to the expansile intraoperative descending aortic dissection observed from TEE, this is the rare case of TEVAR following TAVR. It should be noted that the causes of early death after TAVR.

There are two choices of anesthesia for consideration in TAVR.^16^ In the early years of TAVR, general anesthesia was the default option, and then, local anesthesia plus sedation started to be conducted. In one systemic review,^17^ local anesthesia plus sedation was reported to be more beneficial for hemodynamic stability and the duration of hospitalization. Moreover, the advantage of local anesthesia would be the patient being able to report pain if there is aortic injury. However, it did not impact the 30‐day mortality. In terms of the detection of aortic dissection complication, general anesthesia enables the use of TEE, which can theoretically assist in optimal valve deployment and facilitate the recognition of complications, whereas local anesthesia plus sedation cannot.

## 4 CONCLUSION

Although immediate aortic dissection is an unexpected and rare complication, especially descending aortic dissection from post‐TAVR, it can lead to death if it occurs. To prevent immediate vascular complications going undiagnosed, we suggest interventionists could do TEE in every patient after TAVR when there is any doubt of aortic complication before transferring the patient to the recovery room, so that any complication that may occur will be checked and treated in time.

## CONFLICT OF INTEREST

None declared.

## AUTHOR CONTRIBUTIONS

PR and AJ: collected and provided all the patient data and imaging. PR and AJ: reviewed and drafted the manuscript. DJ, AM, NW, NC, and PS: made critical revisions to the manuscript. All the authors read and approved the final manuscript.

## CONSENT FOR PUBLICATION

Verbal and written consent for publication was obtained from the patient.

## Data Availability

The data that support the findings of this study are available from the corresponding author upon reasonable request.
